# Complexity Analysis of Iterative Basis Transformations Applied to Event-Based Signals

**DOI:** 10.3389/fnins.2018.00373

**Published:** 2018-06-12

**Authors:** Sio-Hoi Ieng, Eero Lehtonen, Ryad Benosman

**Affiliations:** ^1^INSERM UMRI S 968, Sorbonne Universites, UPMC Univ Paris 06, UMR S 968, Centre National de la Recherche Scientifique, UMR 7210, Institut de la Vision, Paris, France; ^2^Department of Future Technologies, University of Turku, Turku, Finland

**Keywords:** event-based signal processing, AER, discrete basis transforms, DWT, DCT

## Abstract

This paper introduces an event-based methodology to perform arbitrary linear basis transformations that encompass a broad range of practically important signal transforms, such as the discrete Fourier transform (DFT) and the discrete wavelet transform (DWT). We present a complexity analysis of the proposed method, and show that the amount of required multiply-and-accumulate operations is reduced in comparison to frame-based method in natural video sequences, when the required temporal resolution is high enough. Experimental results on natural video sequences acquired by the asynchronous time-based neuromorphic image sensor (ATIS) are provided to support the feasibility of the method, and to illustrate the gain in computation resources.

## 1. Introduction

Linear basis transformations are some of the most widely applied mathematical operations in image and signal processing. The main reason in using them is to find adequate bases in which specific properties of a signal are made easier to extract. Variations of the Fourier transform (e.g., the Discrete Fourier Transform (DFT) and the Discrete Cosine Transform) and wavelet transformations are important examples of the omnipresent techniques—popularized by digital standards such as JPEG or JPEG2000—to achieve signal filtering and compression. The basis transformations are well established through decades of research in signal processing and are applied successfully to modern digital image and video processing using the frame-based representation. The most successful basis transform developed for image and general signal processing is without a doubt the Fast Fourier Transform (FFT) where the Cooley-Tuckey algorithm (Cooley and Tukey, 1965) is the common form used for computing the FFT. The complexity of this optimized algorithm is O(nlog(n)), which is significantly lower than that of the direct computation using the Fourier transform's mathematical definition. In order to capitalize the complexity gain obtained by using the Cooley-Tuckey algorithm, many dedicated hardware realizations have been designed for real time computing applications, for example (Baas, 1999; Lin et al., 2005; Uzun et al., 2005). The Fourier transform is known to be an inappropriate technique for capturing transient frequencies in temporal signal analysis. The short term Fourier transform was introduced to study signals with frequencies that change over time, and the wavelet transforms were introduced to capture local changes in time and/or in space at various scales in a seamless way (Sejdic et al., 2009). This property makes the wavelet transforms highly useful for example in natural signal processing, prediction and compression. Dedicated hardware implementations of Discrete Wavelet Transforms (DWT) are numerous; for example (Edwards and Cauwenberghs, 1995; JPEG2000, 2017). From the perspective of the presented work, we note that these conventional implementations of the basis transforms are computationally efficient in the frame-based context. However, they are not directly applicable to event-based signals and in a naive implementation require the synthesis of “virtual” frames, which in turn results in the loss of many advantageous properties of the event-based signals such as the high temporal resolution and the temporal redundancy suppression. The transformation theories and algorithms on which this work is referring to are mainly (Cooley and Tukey, 1965; Daubechies, 1992; Sweldens, 1996) since they established the foundation of the modern signal transformations algorithms.

The first embodiment of neuromorphic sensing appeared in the 1990's in the form of a silicon retina (Mahowald and Mead, 1991). In contrast to standard imaging technology, the neuromorphic imaging mechanism is based on the concept of “events” which are asynchronous and sparse. Since then, the neuromorphic vision sensors have advanced with several generations of the dynamic vision sensor (DVS) (Lichtsteiner et al., 2008) and the asynchronous time-based image sensor (ATIS) (Posch et al., 2011), which captures the relative changes and also the absolute light intensities for further processing. The captured visual information is encoded as events using the address event representation (AER). This representation is fundamentally different from the frame-based one, and thus the basis transformation operations need to be reformulated for event-based signals.

In this work we present a general methodology for computing efficiently arbitrary linear transformations on event-based signals. The paper is organized as follows. Section 1.1 provides an overview of the event-based imaging sensor technology, while section 1.2 discusses event-based information processing. The formalization of the event-based basis transformation is then derived in section 2.1 from the conventional mathematical definitions. Widely used basis transformations and their implementational details are described in section 2.2. Experimental results are presented in section 3 are carried out using natural image data acquired with the ATIS sensor, and the computational performance expressed by the number of multiply-and-accumulate (MAC) operations is measured under different imaging conditions. Finally section 4 discusses the relationship of the presented work and the conventional frame residuals-based video transformation methods.

### 1.1. Event-based imaging

Biomimetic, event-based cameras are a novel type of vision sensors that—like their biological counterparts—are driven by events taking place in the observed scene. This is in contrast to conventional vision sensors, which are driven by artificially created timing and control signals (e.g., frame clock) that have no relation whatsoever to the source of the visual information (Lichtsteiner et al., 2008). Over the past few years, a variety of these event-based cameras has been designed, including temporal contrast vision sensors that are sensitive to relative luminance change, gradient-based sensors sensitive to static edges, edge-orientation sensitive devices, and optical-flow sensors. Most of these vision sensors output visual information about the scene in the form of events using the Address Event Representation (AER) (Mahowald, 1992; Lazzaro and Wawrzynek, 1995; Boahen, 2000) and encode the visual information in the dimension of time instead of voltage, charge or current. The ATIS used in this work is a time-domain encoding vision sensor with 240 × 304 pixels resolution (Posch et al., 2011). The sensor contains an array of fully autonomous pixels that combine an illuminance change detector circuit and a conditional exposure measurement block.

**Figure 1:**
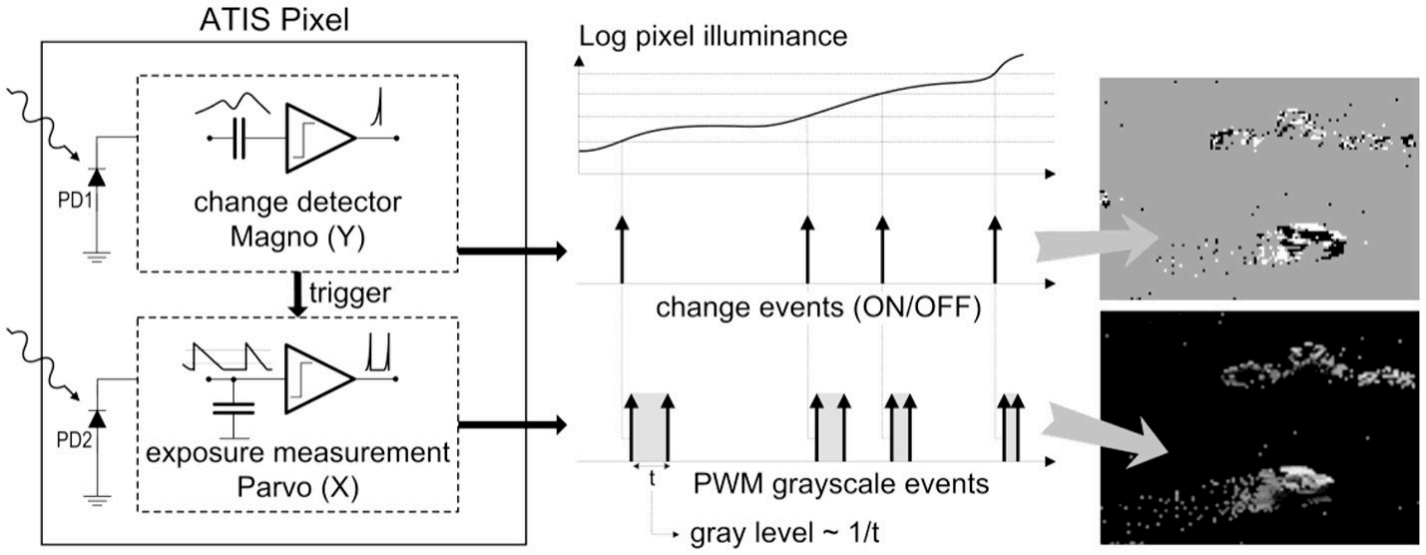
Functional diagram of an ATIS pixel. Two types of asynchronous events, encoding change and brightness information, are generated and transmitted individually by each pixel in the imaging array.

As shown in the functional diagram of the ATIS pixel in Figure 1, the change detector individually and asynchronously initiates the measurement of an exposure/gray scale value only if—and immediately after—a brightness change of a certain magnitude has been detected in the field-of-view of the respective pixel. The exposure measurement circuit in each pixel individually encodes the absolute instantaneous pixel illuminance into the timing of asynchronous event pulses, or more precisely into the inter-event time intervals.

Since the ATIS is not clocked like conventional cameras, the timing of events can be conveyed with a very accurate temporal resolution at the order of microseconds. The time-domain encoding of the intensity information automatically optimizes the exposure time separately for each pixel instead of imposing a fixed integration time for the entire array, resulting in an exceptionally high dynamic range and improved signal to noise ratio. The pixel-individual change detector allows to reduce largely temporal redundancies, resulting in a sparse encoding of the image data.

**Figure 2:**
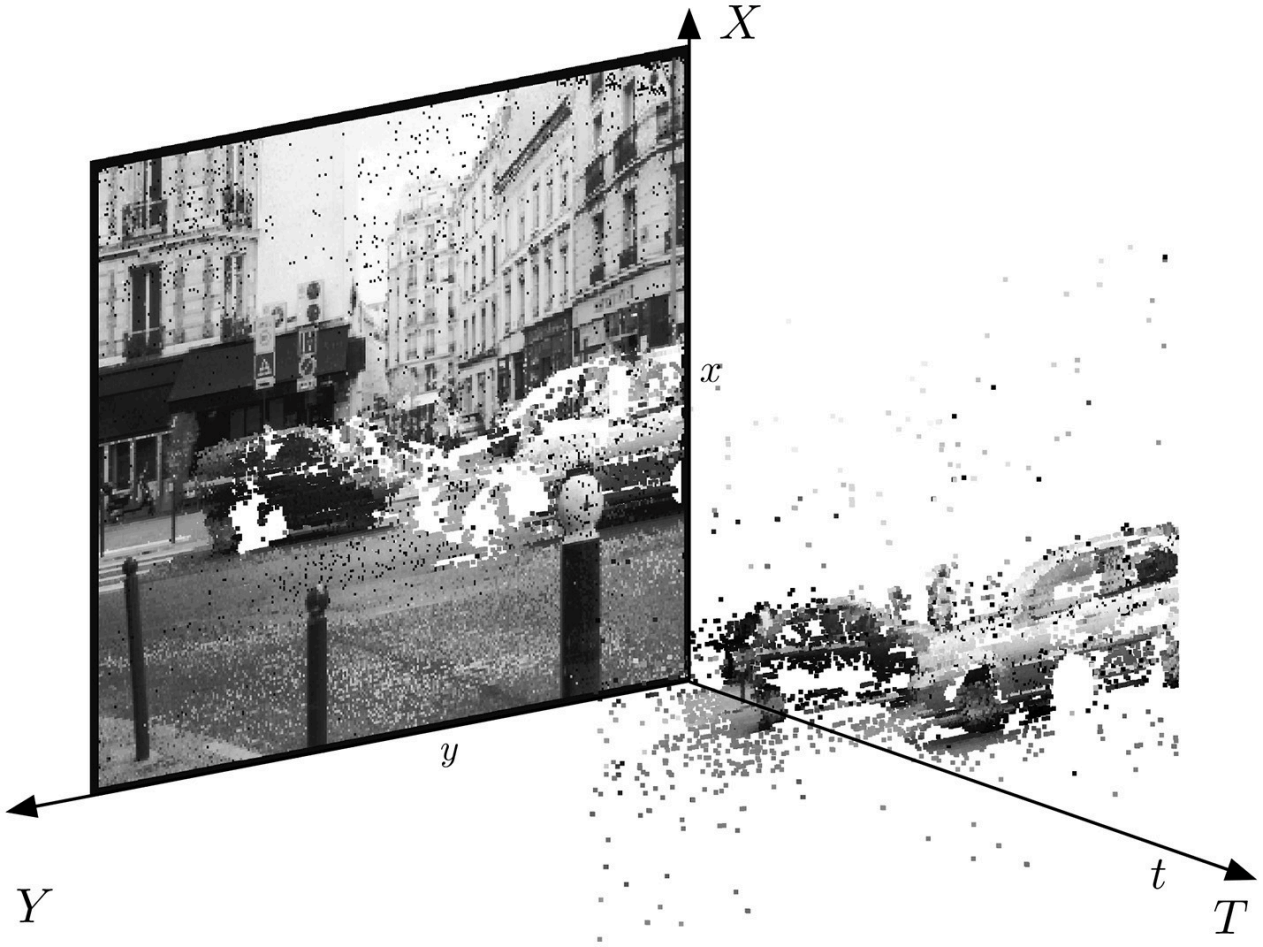
The spatio-temporal space of imaging events: Static objects and scene background are acquired first. Then, dynamic objects trigger pixel-individual, asynchronous gray-level events after each change. Frames are absent from this acquisition process. Samples of generated images from the presented spatio-temporal space are shown in the upper part of the figure.

Figure 2 shows the general principle of asynchronous imaging spaces. Frames are absent from this acquisition process. They can however be reconstructed, when needed, at frequencies limited only by the temporal resolution of the pixel circuits (up to hundreds of kiloframes per second). Static objects and background information, if required, can be recorded as a snapshot at the start of an acquisition; henceforth, the moving objects in the visual scene describe a spatio-temporal surface at a very high temporal resolution. In the following we will present a general way to apply linear transformations on the change detector events.

### 1.2. Event-based signal processing

The AER used in the silicon retina encodes visual information as spatio-temporal events instead of a sequence of frames. This introduces a new paradigm in computer vision. Research on processing techniques suitable for AER has been prolific since these past few years, and several results have been achieved in the use of the silicon retinas. An interesting fact on most of previously published works is the exclusive use of change events to extract useful information from the scene. One reason for this is that former silicon retinas were able to output only change events. Direct translations of state of the art computer vision algorithms are usually achieved by using the illuminance information estimated by local integration of the change events. This approach is adopted by several previous works, for example in using event correlation for stereomatching (Kogler et al., 2011), in photoconsistency based optical flow (Benosman et al., 2012), and in machine learning using convolution networks (Perez-Carrasco et al., 2013). The second reason to use only change events is that, for most of machine vision problems, time is proven to be an information medium that substitutes surprisingly well for illuminance. Stereovision reformulated for the asynchronous silicon retinas is an interesting example showing that classic projective geometry combined with a high temporal accuracy provide an accurate criterion for matching events and triangulating 3D structures (Rogister et al., 2011; Carneiro et al., 2013). Tracking algorithms that take advantage of the accurate timing have been developed for event-based visual signals: the event-based reformulation of Hough-transform based circle tracker in Ni et al. (2011), the iterative algorithm for tracking predefined shapes (Ni et al., 2015), and the part-based tracking technique in Reverter-Valeiras et al. (2015) are a few examples of event-based tracking algorithms that require little computations upon the arrival of each new event. Time as the main information medium is emphasized with HFirst (Orchard et al., 2015), the hierarchical model of the visual cortex derived from the HMAX (Riesenhuber and Poggio, 1999). It demonstrates that visual learning can be achieved through temporal information.

This list of event-based signal processing algorithms, while not comprehensive, gives an overview of the state-of-the-art event-based visual signal processing methods. As mentioned above, these algorithms process only change events. Only a handful of studies dealing directly with the event-based illuminance (encoded as gray-levels) can be listed so far from the literature. A compressive sensing reconstruction of the illuminance has been implemented on hardware in Orchard et al. (2012). The idea behind it is to exploit the stochastic false change detection due to noise in the ATIS. The high temporal accuracy of the sensor is then traded off to reconstructing the missing illuminance information, and as a result, 28 Hz videos achieving state-of-the-art visual quality are obtained. In Ieng et al. (2014) asynchronous linear and non-linear filters have been developed for generalizing image filtering techniques to event-based gray-levels. The illuminance information is a supplementary visual information for the above listed algorithms, but it is a mandatory information for displaying event-based signal in a realistic and human-friendly way. The use of illuminance information is a step toward a unified formulation of visual signal processing that encompasses both frame-based and event-based representation. Using such an approach one can tackle spatial frequency analysis, image compression and even high dynamic range imaging that are heavily relying on the illuminance information, and explore the impact of the integration of illuminance information to the event-based signal processing.

One important observation should be emphasized about the present paper is the context of this work that focuses exclusively proposing an iterative, event-by-event adaption of the classical basis transformations. A complexity analysis is provided to show the inherent possibility to save computation power thanks to the low redundancy of the event-based signal to process. The problem of sparse representation has been widely tackled by the communities of adaptative and compressive sensing, the main concern of these domains is the initial signal reconstruction from one sparse basis to another one (Candes et al., 2006; Vaswani, 2008). This is however a totally different problem that we are not aiming to step in as signal reconstruction is an extremly costly offline processing. Rather, in this work we are aiming to provide an easy to implement and computationaly cheap event-based algorithm that can process events provided by an event-based sensor on the fly.

## 2. Materials and methods

### 2.1. Event-based basis transformation

#### 2.1.1. General formulation

The event-based representation assumes that only a few pixels change at a given time, implying only local updates of the signal content. To simplify the notations, and without loss of the problem's generality, we assume that at a given time only the *i*th pixel changes its value from *x*_*i*_ to ${\hat{x}}_{i}$; multiple-pixel updates are then performed by applying single pixel updates on these pixels sequentially.

**Figure 3:**
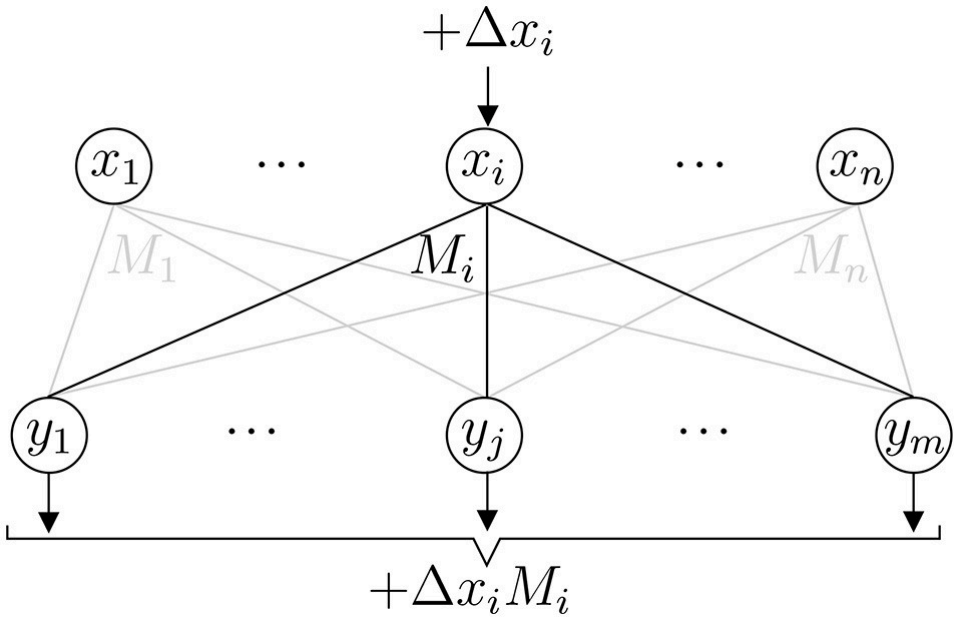
Event update step. A change in *x*_*i*_ changes the output **y** by Δ*x*_*i*_*M*_*i*_.

Let us first consider a one-dimensional sensor whose output **x** is a column vector of length *m*. In the following we investigate linear transformations of the form *f*:𝕂^*m*^ → 𝕂^*n*^, where 𝕂 is either ℝ or ℂ, and where *m* and *n* are the dimensions of the considered vector spaces. Each linear transform *f* can be represented by a matrix *M* for which

(1) $$\begin{array}{l} \text{y}=M\text{x}, \end{array}$$

where **x** corresponds to the current values of the pixels, and **y** is the value of the transform. Let us write *M* in a column vector form *M* = (*M*_1_, …, *M*_*m*_). We denote by $\hat{\text{x}}$ the updated vector, where the pixel that has been updated is denoted by ${\hat{x}}_{i}$, and similarly by **x** and *x*_*i*_ the pixels before the single update. The output of the linear transform before and after the transform, respectively, is denoted by **y** and $\hat{\text{y}}$. Then

(2) $$\begin{array}{l} \hat{y}=M\hat{x}=M(x+(\hat{x}-x))\\ =Mx+M\left(\left(\begin{array}{c} x_{1}\\ .\\ {\hat{x}}_{i}\\ .\\ x_{n} \end{array}\right)-\left(\begin{array}{c} x_{1}\\ .\\ x_{i}\\ .\\ x_{n} \end{array}\right)\right)\\ =y+(M_{1},\ldots ,M_{i},\ldots ,M_{m}){\left(0,\ldots ,{\hat{x}}_{i}-x_{i},\ldots ,0\right)}^{T}\\ =y+({\hat{x}}_{i}-x_{i})M_{i}\\ =y+\Delta x_{i}M_{i}, \end{array}$$

where $\Delta x_{i}={\hat{x}}_{i}-x_{i}$ is the amount by which the *i*th pixel has changed (Figure 3). On the whole then

(3) $$\begin{array}{l} \Delta \text{y}=\hat{\text{y}}-\text{y}=\Delta x_{i}M_{i}, \end{array}$$

where *M*_*i*_ is the *i*th column of the transform matrix *M*. Since there are *n* elements in *M*_*i*_, this event update rule for **y** takes *n* multiply-and-accumulate (MAC) operations. For convenience, as is the typical case in image transformations, we consider in the following the case *m* = *n*. Then applying this update rule for every sensor element once takes *n*^2^ MACs, which is the same number of MACs that is required in applying the matrix multiplication $M\hat{\text{x}}$ directly, when *M* is a *n*×*n* matrix. This shows that the event update rule (3) does not introduce overhead in the computations for a general linear transform.

This mechanism can be generalized to more complex and non-linear transforms if the assumption of infinitesimal changes of *x*_*i*_ holds (i.e., Δ*x*_*i*_ ≈ 0). In such a case, we can use a first order approximation to update **y**:

(4) $$\begin{array}{l} \hat{\text{y}}\approx \text{y}+J_{f}\left(\text{x}\right)\left(\text{x}-\hat{\text{x}}\right), \end{array}$$

where *J*_*f*_(**x**) is the Jacobian matrix of *f* at **x** and the first order term in (4) is the vector

(5) $$\begin{array}{l} \left(J_{1}\left(\text{x}\right),\ldots ,J_{m}\left(\text{x}\right)\right)\left(\text{x}-\hat{\text{x}}\right)=\Delta x_{i}J_{i}\left(\text{x}\right), \end{array}$$

according to (3), where we set *M* = *J*_*f*_.

#### 2.1.2. Event-based linear 2D transform

Let us generalize the discussion above to 2D signals, that is, instead of vectors of length *m* we assume that the sensor outputs a matrix *X* of size *m*×*n*. Similarly to the above, $\hat{X}$ is the updated matrix, and ${\hat{x}}_{i,j}$ the value of the updated pixel at location (*i, j*). Let us consider transformations of the form

(6) $$\begin{array}{l} Y=UXV, \end{array}$$

where *U* is a *k*×*m* matrix and *V* is an *n*×*l* matrix, and therefore *Y* is a *k*×*l* matrix, where *n*, *m*, *k*, and *l* are natural numbers. Many practically important 2D transformations — such as the Fourier transform, the discrete cosine transform (DCT), and (DWT) — can be written in this form.

Let us denote *W* = *UX*. Then the first update step corresponding to $W\mapsto \hat{W}$ is achieved similarly to (3). If *W* is written in the column form *W* = (*W*_1_, …, *W*_*n*_), then

(7) $$\begin{array}{l} \{\begin{array}{c} {\hat{W}}_{t}=W_{t}\;\;\forall t\neq j,\;\text{and}\\ {\hat{W}}_{j}=\Delta x_{i,j}U_{i} \end{array} \end{array}$$

where (*i, j*) is the coordinate of the updated pixel and *U*_*i*_ is the *i*th column of *U*. In other words, the event update changes only the *j*th column of *W*, and thus this step requires *k* MACs.

The second step of the transform performs the update of *Y* according to $\hat{Y}=\hat{W}V$. As noted above, $\hat{W}$ and *W* coincide except at the *j*th column, and therefore

(8) $$\begin{array}{l} \hat{Y}=\left(W+\left(\hat{W}-W\right)\right)V\\ =WV+\left(0,\ldots ,{\hat{W}}_{j},\ldots ,0\right)V\\ =Y+{\hat{W}}_{j}V^{j}\\ =Y+\Delta x_{i,j}U_{i}V^{j}, \end{array}$$

where *V*^*j*^ is the *j*th row of *V*. Thus in general this 2D transform requires *k*+*kl* MACs, as the outer product takes *kl* MACs.

In the following we concentrate on image transforms for which *k* = *l* = *m* = *n*, and thus a 2D event update requires in general *n* + *n*^2^ MACs. Moreover, the transforms we consider satisfy *V* = *U*^*T*^, and thus the update rule becomes

(9) $$\begin{array}{l} \hat{Y}=Y+\Delta x_{i,j}U_{i}U_{j}^{T}. \end{array}$$

Now, if *U* is a sparse matrix that has at most *s* non-zero elements per column, the 2D event update takes at most $s+s^{2}=O\left(s^{2}\right)$ MACs. This observation will be useful as we consider wavelet transforms in section 2.2.2, and show that these transforms are particularly efficient for performing the event update rule.

#### 2.1.3. Clusters of events

The event-based formulation assumes the processing of the data on arrival of each individual event in a sequential manner, however Equation (9) is extendable to events that occur at the same time in an almost straightforward manner. Let us assume the set of N events σ = {(*i, j, t*)}, that occur at the same time *t*. The update equation is the finite sum of the *N* events contributions:

(10) $$\begin{array}{l} \hat{Y}=Y+\underset{\left(i,j\right)\in \sigma }{\sum }\Delta_{i,j}U_{i}U_{j}^{T}. \end{array}$$

As such, the number of MACs is still increasing linearly with the number of events in the set, hence the global complexity is unchanged. However, by extending to a set of simultaneous events, we are actually getting away from the event-based hypothesis and get closer to frame representation. A strategy to switch to fast and optimized classic transformations (FFT,…) when they perform better is necessary.

### 2.2. Important 2D transforms

In the following we apply the results presented in section 2.1 to discrete Fourier-related transforms and wavelet transforms, and compare the computational efficiency of these transforms when the event update rule (9) is used. For convenience of notation, we assume that the input matrix is of size *n* × *n*, where *n* = 2^*k*^ for some *k* ∈ ℕ.

#### 2.2.1. Fourier-related 2D transforms: discrete Fourier and cosine transforms

##### 2.2.1.1. Discrete fourier transform (DFT)

Equation (9) can be naturally applied to reformulate the 2D DFT, whose matrix *U* equals

(11) $$\begin{array}{l} U\left(i,j\right)=\left(1/\sqrt{n}\right)\omega^{\left(i-1\right)\left(j-1\right)}, \end{array}$$

for *i, j* = 1, …, *n*, and $\omega =e^{\left(-2\pi \sqrt{-1}\right)/n}$.

Thus *U* is a dense matrix, which implies that *n*+*n*^2^ (complex) MACs are required to update the transform upon arrival of one single event.

##### 2.2.1.2. Discrete cosine transform (DCT)

For the discrete cosine transform the transform matrix *U* equals

(12) $$\begin{array}{l} U\left(i,j\right)=\cos\left(\frac{\pi \left(j-1/2\right)\left(i-1\right)}{n}\right), \end{array}$$

for *i, j* = 1, …, *n*. Again, *U* is a dense matrix, and thus the 2D event update step (9) takes *n*+*n*^2^ (real) MACs.

#### 2.2.2. 2D discrete wavelet transforms

**Figure 4:**
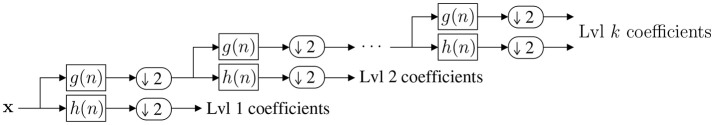
Computation of the discrete wavelet transform using consecutive filtering and down-sampling by two. Here *h*(*n*) denotes the high-pass filter and *g*(*n*) denotes the low-pass filter of the wavelet transform. Using this approach, the Haar transformation of a *n*×*n* image takes approximately (16/3)*n*^2^ MACs, as a single level of the transform takes 4*n*^2^ MACs and as the Wavelet transform is applied recursively to the upper left corner of the transformed image.

A 2D DWT of the signal *X* is a linear transform that can be written in the matrix form *Y* = *HXH*^*T*^, where *H* and *H*^*T*^ are the column and the row transform matrices, respectively. In conventional signal processing this form is not computationally the most efficient, and the wavelet transform is preferably implemented with the filter bank approach as introduced in Mallat (1989). The filter bank structure is depicted in Figure 4 and is a succession of *k* blocks, where each block applies high-pass filtering (here denoted by *h*), low-pass filtering (denoted by *g*), and downsampling by two (denoted by ↓2) on the signal obtained from the previous level of the filter bank structure. While the matrix multiplication scheme requires more MACs in the frame-based approach than the filter-bank approach does, we will show that it is an efficient way to perform the wavelet transform using the event-based update Equations (3) and (8).

#### 2.2.2.1. Haar wavelet transform

Let us first consider the Haar wavelet transform, which is one of the most important wavelet transforms due to its simplicity. Its low-pass and high-pass filters *g* and *h* are defined by the coefficients (1, 1) and (1, −1). As explained in Appendix 0.1, up to a normalization of the rows, the transform matrix *U* = *H* can be built through the recursive rule :

(13) $$\begin{array}{l} \forall i\in {\mathbb{N}}^{*},H_{2m\times 2m}=\left(\begin{array}{c} H_{m\times m}⊗\left(1,1\right)\\ I_{m}⊗\left(1,-1\right) \end{array}\right), \end{array}$$

where $H_{2\times 2}=\left(\begin{array}{cc} 1 & 1\\ 1 & -1 \end{array}\right)$. The number of non-zero elements per column of *H*_*n*×*n*_ is *s* = log_2_(*n*)+1 (see Appendix 0.1 for the proof), and thus by (3), the number of MACs required by a single update in a vector of size *n* is log_2_(*n*)+1, not *n* as in the general case. The number of MACs required by the 2D transform update step (9) is

(14) $$\begin{array}{l} s^{2}+s={\left(\log_{2}\left(n\right)+1\right)}^{2}+\left(\log_{2}\left(n\right)+1\right)\\ ={\left(\log_{2}\left(n\right)\right)}^{2}+3\log_{2}\left(n\right)+2. \end{array}$$

The number of bits needed to store *H* is O(nlog(n)), since *H* is sparse, and the number of MACs required by the event update step is $O\left(\log{\left(n\right)}^{2}\right)$, which compares favorably to the $O\left(n^{2}\right)$ MACs required by the update step of a general dense 2D linear transformation. This complexity reduction is not only due to the event-by-event processing of the data but also because of the sparse structure of *H*. This is also benefiting frame-based calculation of the wavelet transform.

#### 2.2.2.2. General wavelet transform

For a general DWT, there is no obvious iterative way based on the Kronecker product to build the matrix *H*. However, *H* has a general structure that can be used to determine an upper bound to the number of non-zero elements per column. In this subsection, we analyze the structure of the matrix *H* and derive an a upper bound to the number of non-zero element in each of its columns.

**Figure 5:**
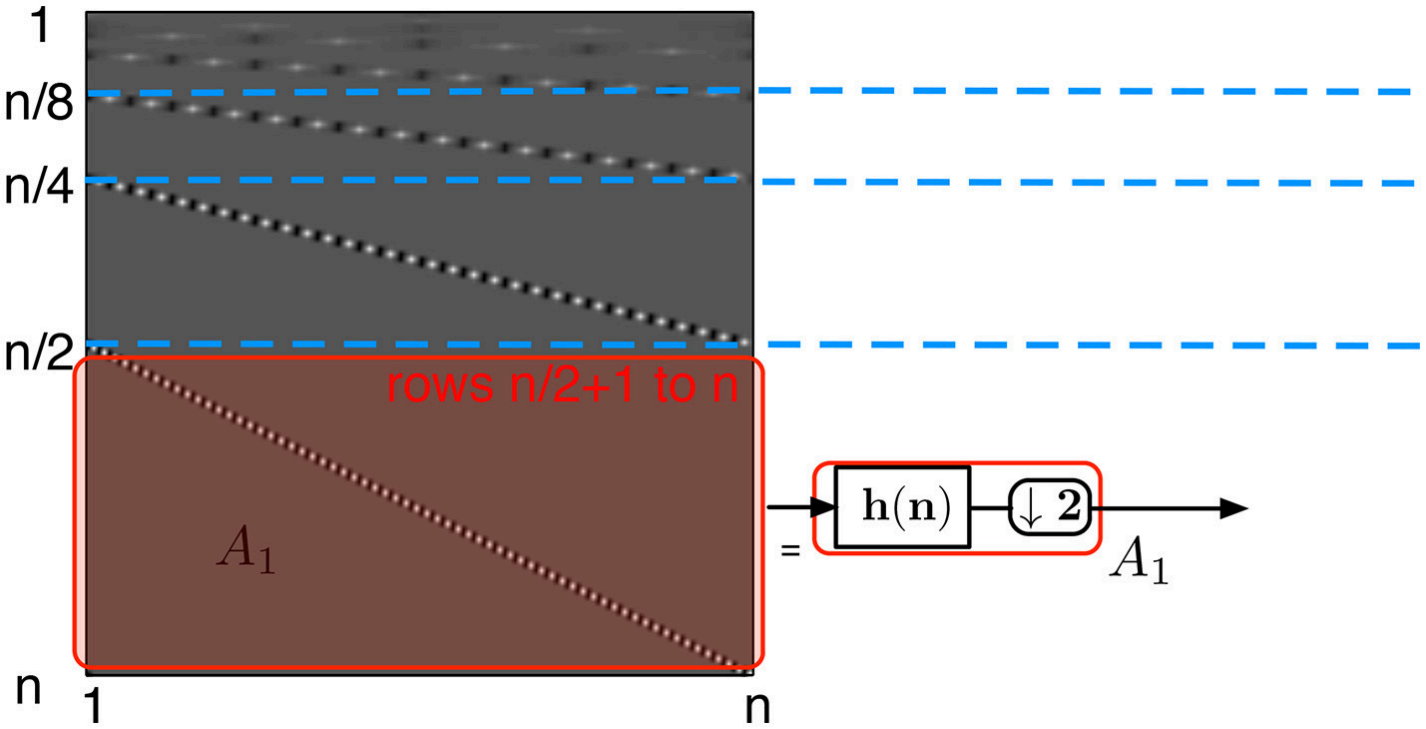
Wavelet transform matrix at the first level. The left structure is a schematic of the transform matrix split into submatrix *A*_*i*_. The first step is represented by the bottom half of the matrix, *A*_1_ and corresponds to a high-pass filter followed by the downsampling by two elements.

**Figure 6:**
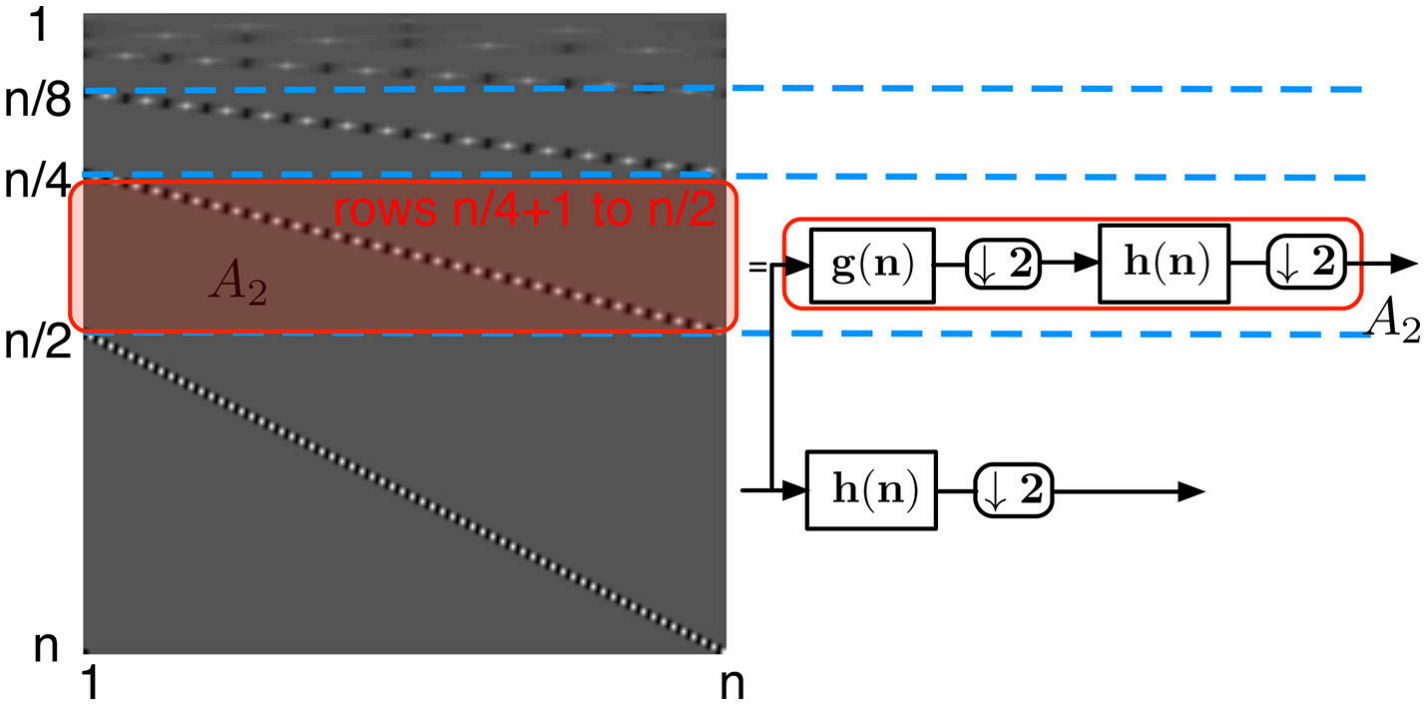
Wavelet transform matrix at the second level. The submatrix defined from row *n*/4+1 to row *n*/2, *A*_2_, corresponds to consecutive low-pass filtering, downsampling by 2, high-pass filtering, and again downsampling by two elements.

**Figure 7:**
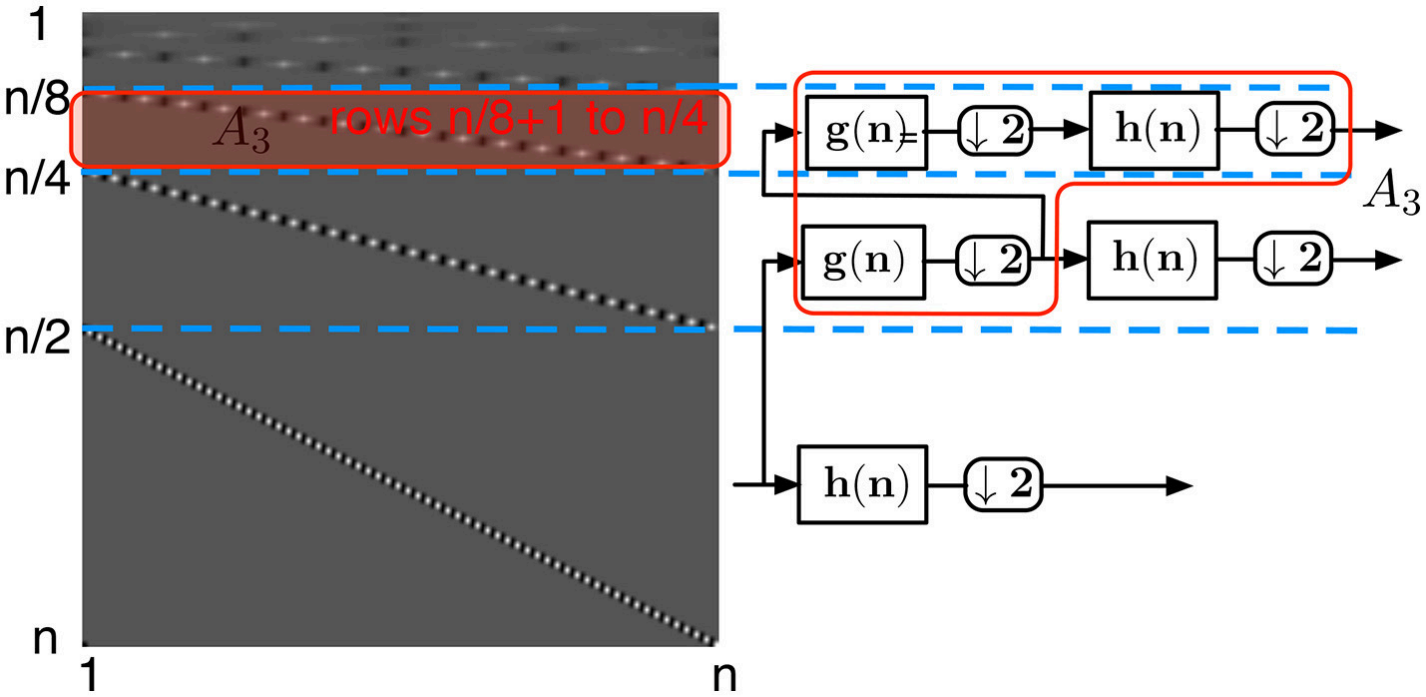
Wavelet transform matrix at the level *i* = 3.

Let us denote by *h* and *g* the finite impulse response filters of the considered DWT. Let us assume that *h* and *g* contain only non-zero coefficients, and let *p* the length of the longer of these two filters. Furthermore, let *l* be the smallest integer that satisfies *p*/2 ≤ 2^*l*^. The transform matrix *H* has then the following structure:

- each of the rows of the submatrix *A*_1_ (Figure 5)—defined from row $\frac{n}{2}+1$ to row *n* of *H* — is a circularly shifted copy of the row $\frac{n}{2}+1$, where the circular shift is taken two elements to the right per row. Each of the rows has at most *p* non-zero elements corresponding to the high-pass filter coefficients. Due to the circular shift, the number of non-zero elements per column in this submatrix is at most *p*/2
- the submatrix *A*_2_ (Figure 6) defined from row $\frac{n}{4}+1$ to row $\frac{n}{2}$ corresponds to the second level of coefficients of the wavelet transform. These coefficients are obtained by applying the high-pass filter *h* onto a low-pass filtered and downsampled vector. Due to the convolution of the high-pass and low-pass filters, each row contains at most 2*p* non-zero coefficients, and due to the two separate downsamplings by two, each row is circularly shifted by four steps. Thus again each column of this submatrix contains at most *p*/2 non-zero elements.
- the same observation can be done for each submatrix *A*_*i*_ (Figure 7) defined from row $\frac{n}{2^{i}}+1$ to row $\frac{n}{2^{i-1}}$ of *H*. Again, each of their columns has at most *p*/2 non-zero elements. Notice that the topmost submatrix is defined from row 1 to row 2^*l*^.

Finally, as *H* consists of submatrices *A*_1_, *A*_2_, …, *A*_*k*−*l*+1_, the total number of non-zero elements in each of its columns is at most

(15) $$\begin{array}{l} C\left(p,n\right)=\left(p/2\right)\left(\log_{2}\left(n\right)-l+1\right), \end{array}$$

with *k* = log_2_(*n*).

For example, the result for the Haar transform is obtained with *p* = 2, which implies *l* = 0 and *C*(2, *n*) = log_2_(*n*)+1. For the Daubechies 5/3 transform matrix, *p* = 5, and thus *l* = 2 and *C*(5, *n*) = (5/2)(log_2_(*n*) − 1).

**Figure 8:**
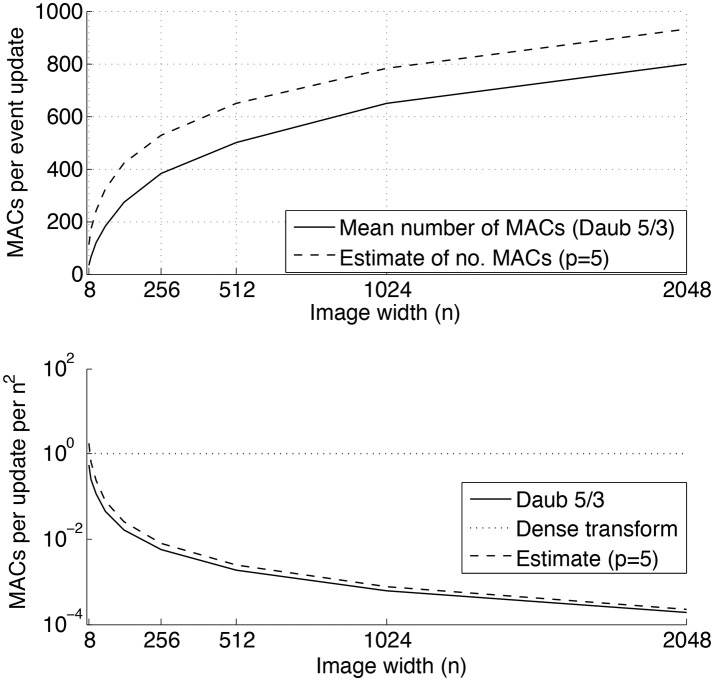
Number of MACs required by a single event update of a 2D Daubechies 5/3 discrete wavelet transform. **Top inset:** mean number of MACs required by a single event update, and an upper bound estimation using (15) with *p* = 5. **Bottom inset:** Number of MACs required by a single event update per the total number of pixels *n*^2^. As can be seen, the discrete wavelet transform is much more efficient in terms of numbers of MACs than a general dense transform.

From this observation, we can conclude that for any DWT, the event update step (7) requires $O\left(\log_{2}\left(n\right)\right)$ MACs, and hence the 2D event update step (9) requires $O\left(\log_{2}{\left(n\right)}^{2}\right)$ MACs. The generalization to a *m*×*n* transform matrix *M* is straightforward: (7) and (9) are requiring respectively $O\left(\log_{2}\left(m\right)\right)$ and $O\left(\log_{2}\left(m\right)\log_{2}\left(n\right)\right)$ MACs to update the transform. An estimate of the computational complexity can be sketched by counting the number of MACs required by a single update of the Daubechies 5/3 DWT (Daubechies, 1992). In Figure 8, the top inset shows the number of MACs per an event update with respect to the increasing size, *n*×*n*, of the input image for *n*∈[8, 16, 32, …, 2048]. In the bottom inset the number of MACs per an event update, normalized by the total number of pixels *n*^2^, is compared to a dense basis transform. As can be seen, the normalized number of MACs decreases with *n* for the wavelet transform, while this ratio remains constant for the dense transform.

The MACs estimation for the standard transforms are established assuming dense and non-symmetric transform matrices in general. For specific transforms such as Fourier, symmetry can be exploited to produce fast and efficient algorithms such as the FFT. For transformations like the wavelets' ones, the sparsity is an additional property that should be taken into account. While we only compare the event-based approach with the classic filterbank architecture, it would have been fairer to compare with the improvement introduced in Daubechies and Sweldens (1996) by the lifting scheme. However this is not changing fundamentally the results shown in the next section since as reported in Daubechies and Sweldens (1996), the complexity of the lifting scheme is still linear and the number of operations can be reduced up to half of what is needed for the classic filterbank technique.

More complex optimization techniques can help in reducing frame-based wavelet computation: in Andreopoulos and van der Schaar (2008), an incremental wavelet computation is introduced to exploit the idea that a non-exact transform is acceptable if the induced distortion is limited. This strategy is based on finding a compromise between the transformation accuracy and the resource allocated to compute the transformation. In the extension of the analysis we are doing here, we can imagine to integrate that mechanism also into the event-based update and stop the calculation when signal distortion is below some threshold.

Finally, in a more general context of basis transformation, for frame-based transform, a mechanism of detecting sparsity in the input can be used to either decide to run an actual recalculation on the input when the input is actually not sparse.

## 3. Results

**Figure 9:**
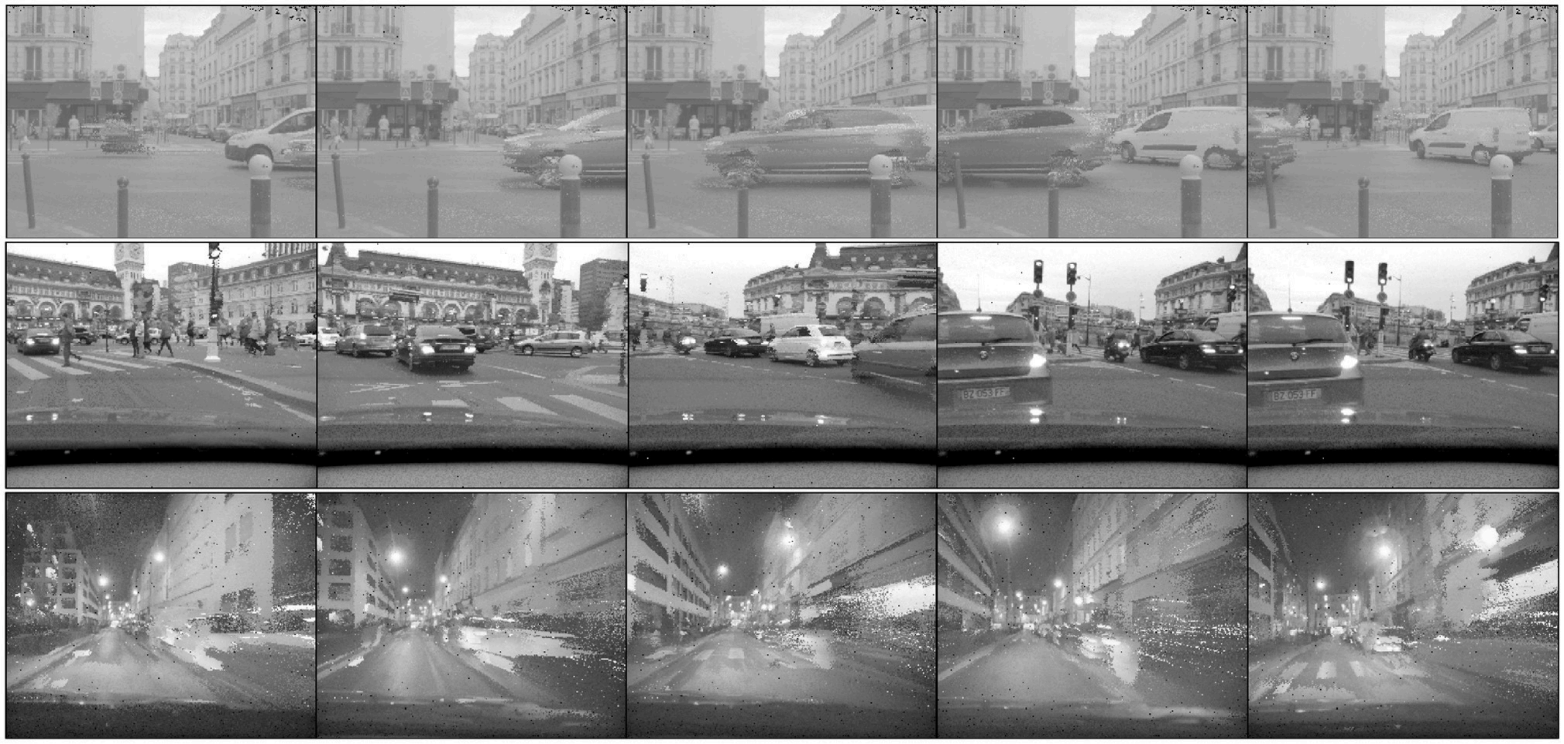
Samples of frames generated from the ATIS: three natural and dynamic scenes with different lighting condition are used for evaluating the event-based basis transform. **(Top)** A sequence of street traffic with the static camera. **(Middle)** Daytime driving with the camera. **(Bottom)** Nighttime driving with the camera.

In this section we consider the application of the presented event-based transform method to the real signal output of the silicon ATIS retina. The output signal of this sensor is an illuminance value I at (*x, y*), at time *t*, where the temporal resolution is in the order of a few μ*s* in contrast to the considerably slower refresh rate of conventional digital cameras. The theory presented in this paper allows to computationally efficiently apply linear transformations on this illuminance signal, as demonstrated in the following. Three sequences are tested with the DWT: the first sequence, “city traffic” is recorded with a static ATIS observing street traffic while the sequences “city day” and “city night” are captured during different times of the day by the same sensor mounted in a moving car. A set of snapshots generated from the recordings is shown in Figure 9 to demonstrate the input signal used to test the event-based transform method.

To evaluate the computational complexity of the presented event-based transformation method against more conventional frame- based methods, we use the following notations:

- An asynchronous signal acquired by the ATIS has a total number of events *N* and a duration of *T* seconds. The same signal is sampled into a sequence of frame at *F* frame per second (fps). The sequence has thus in total *FT* frames.
- **The number of MACs for the iterative form:** one event triggers the iterative transform (9), and thus it requires *n* + *n*^2^ MACs. For a signal of *N* events, then the number of MACs is *N*(*n* + *n*^2^)
- **The number of MACs for a sequence of** *FT* **frames:** a standard 2D transform as defined in (6) requires two successive matrix-multiplications, each of which requires *n*·*n*^2^ = *n*^3^ MACs per frame. For a *FT* frames sequence, the number of MACs is then 2*FTn*^3^.

Let us denote by R the ratio of the number of MACs required by the event-based transformation and the frame-based one. Then

(16) $$\begin{array}{l} R=\frac{N\left(n+n^{2}\right)}{2FTn^{3}}. \end{array}$$

**Figure 10:**
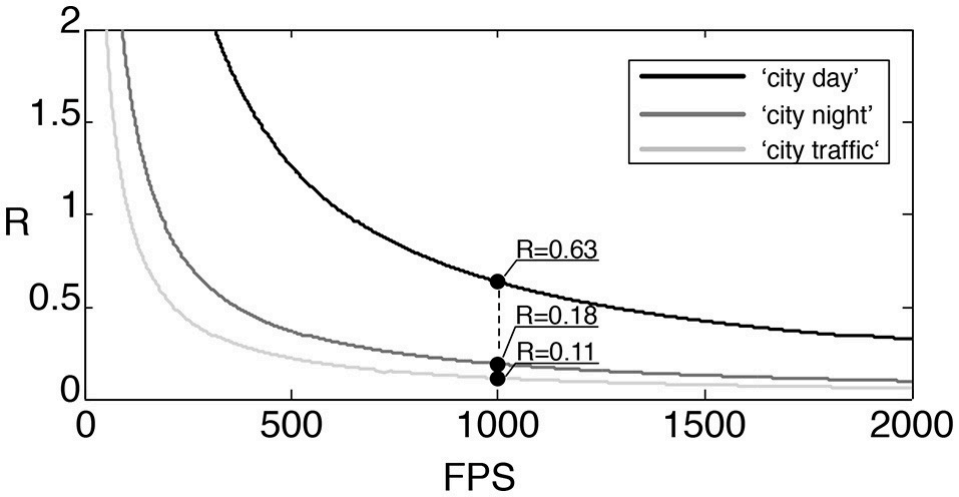
Ratio of MACs used by an iterative transform (DFT, DCT,…) applied to the 3 sequences of outdoor traffic scenes: “city traffic” is a camera static scene while the “city day” and “city night” are acquired with the ATIS embedded in a moving car. At 1,000 fps, the ratio of MACs for the 3 sequences is at most 0.63.

Figure 10 shows the ratio function as defined in (16) for the considered three input sequences. We cropped the signal spatially into a 128 × 128 pixel patch to have spatial dimensions of powers of two in the considered image area. Frames are then generated at an equivalent 1, 000 fps on which we are applying the frame-based transform. A millisecond temporal accuracy is representative of most of natural scenes captured by the asynchronous sensor, which justifies the selection of this frame rate. The ratio *R* is valid for whatever the linear basis change transform as we introduced in the previous sections as long as the transform matrices are dense. As can be seen, in terms of MACs the event-based transform consumes only a fraction of what is required by the dense frame-based transform.

In the above, *R* is defined under the assumption that the basis transform is dense. However, when using wavelet transform, the computational complexities for both event-based and frame-based transformations are significantly reduced as described in subsection 2.2.2. To provide a more accurate comparison for the wavelet transform, we define *R*_*w*_ as the ratio between the number of MACs obtained from using the sparse event-update and the frame-based filter bank method shown in Figure 4. Here we consider the Haar transform, for which the number of MACs per a single event update equals (log_2_(*n*) + 1)^2^ + (log_2_(*n* + 1)), while the transformation of a frame takes asymptotically (16/3)*n*^2^ MACs as described in Appendix 0.2 Therefore

(17) $$\begin{array}{l} R_{w}=\frac{N\left(\log_{2}^{2}\left(n\right)+3\log_{2}\left(n\right)+2\right)}{\left(16/3\right)FTn^{2}}. \end{array}$$

**Figure 11:**
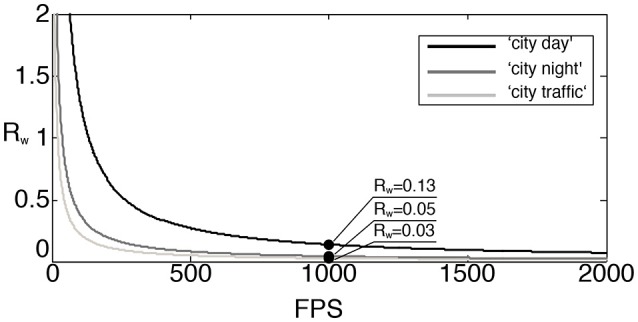
Rectified ratio *R*_*w*_ of MACs in event-based and frame-based wavelet transforms.

The rectified ratio for the wavelet transform with respect to the number of frames per second in the frame-based approach is depicted in Figure 11. At the rate of 1,000 fps, for example, the rectified ratios are much smaller than the ratios *R* at the same fps presented in Figure 10. This increased computational efficiency is due to the exploitation of the sparseness of the transform matrix and the relatively few pixel changes per frame. The “city day” sequence, the more active one in term of recorded events, is the most demanding in computation. Its ratio is now reduced to 0.13 while the other two sequences ratios are lower than 0.05.

At the typical operating frequencies of the asynchronous sensor (several kHz), we can see the event-based transform is much more efficient than the frame-based one, as only updated information needs to be processed. The number of required MACs amounts to 13% of what is used by the filter bank implementation of the Haar transform in the most active sequence. These experiments show that high temporal accuracy signals acquired with the ATIS silicon retina can be exactly transformed in a very resource efficient way when compared to classic state-of-the-art algorithms used in classic image processing.

**Figure 12:**
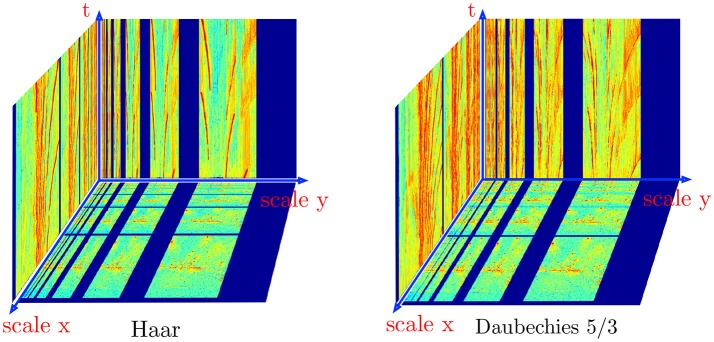
Haar and Daubechies 5/3 wavelet transforms computed iteratively for the night city sequence. The bottom plane is showing the amplitude of the wavelet transforms at an arbitrary time *t*_0_, the transforms are built progressively as graylevel events are output by the ATIS. Slices of the transforms are plotted for visibility reason, the actual structure is a volume of transform coefficients and colors are used to scale the coefficients magnitude.

Finally, for closure of the experimental results, we present in Figure 12 the amplitude of the Haar and Daubechies 5/3 transforms of the “city night” sequence. The results are obtained with the event-based transforms as described above and the complexity of the computation ratio is directly given by rectified ratio in (17). The low and high pass filters coefficients are respectively (up to a normalization factor):

(18) $$\begin{array}{l} \{\begin{array}{l} g_{Haar}=\left(\begin{array}{cc} 1 & 1 \end{array}\right)\\ h_{Haar}=\left(\begin{array}{cc} 1 & -1 \end{array}\right) \end{array} \end{array}$$

and

(19) $$\begin{array}{l} \{\begin{array}{l} g_{Daub5/3}=\left(\begin{array}{ccccc} -\frac{1}{8} & \frac{1}{4} & \frac{3}{4} & \frac{1}{4} & -\frac{1}{8} \end{array}\right)\\ h_{Daub5/3}=\left(\begin{array}{ccc} -\frac{1}{2} & 1 & -\frac{1}{2} \end{array}\right) \end{array} \end{array}$$

This is especially interesting when analyzing the dynamic behavior of a temporal signal. The event-based transform allows to update in a more continuous and less costly way the transient content of the scene. Because the event-based form is an exact reformulation of the standard discrete transforms, there is no need to assess the transformation accuracy.

## 4. Discussion

A compression strategy can be combined to the bank filter technique to reduce even more the computation load of the frame-based transform. By using the differences of images—so-called *image residuals*—it is actually possible to apply the Haar transform only on the pixels that changed between two consecutive frames. This makes the frame-based transformation approach the event-based formalism, when the frame rate increases, thus yielding asymptotically a ratio of MACs close to 1 for these two methods. To verify this, we count the number of MACs required by the bank filter implementation of the Haar transformation applied to image residuals generated at 1, 000 fps. As detailed in the Appendix 0.3, the filter bank implementation of the event-based Haar transform has complexity $O\left(k^{2}\right)$ (where *k* = log_2_*n*), and hence for *N* events, the number of MACs for the transformation amounts to 2*Nk*^2^. In practice this number is usually lower for image residuals which contain spatially clustered pixels, which contribute in updating the same coefficients at each higher wavelet scale. Therefore the exact number of MACs per frame using this image residuals approach depends on the considered video sequence.

**Figure 13:**
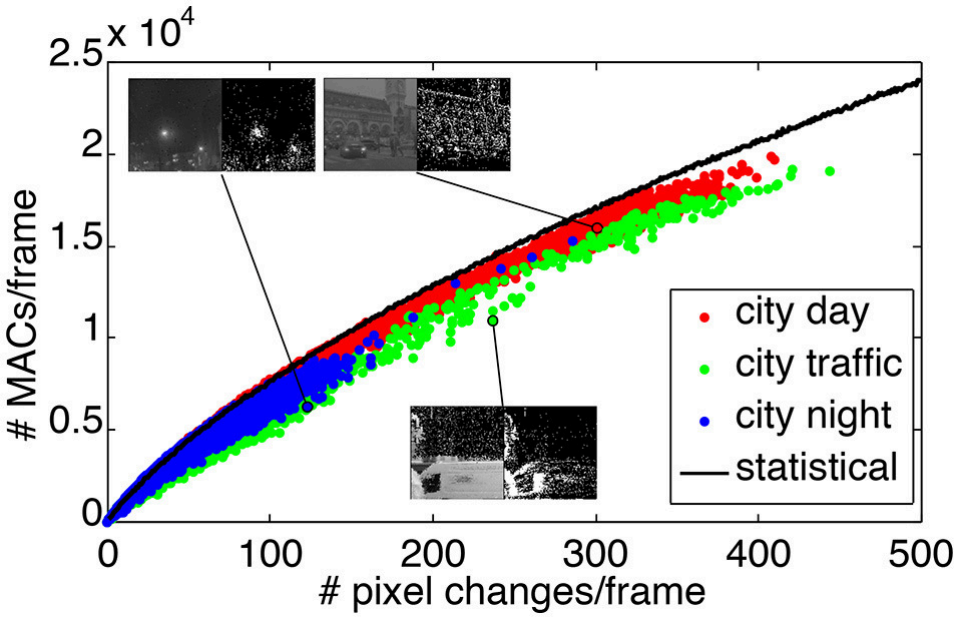
Number of MACs used for bank filter implementation of the Haar transform and applied to the residual images generated at 1,000 fps. The three sequences results are represented by the three color clusters. Each dot is plot as number of MACs vs. the number of non-zero pixel in one residual image. The black curve is the mean number of MACs used for frames that contain an increasing number of pixels that changed but randomly distributed in space. Insets are showing pairs of images and the residuals.

In Figure 13 we present a statistical assessment of the number of MACs per frame required by the filter bank-based wavelet transform applied on image residuals. Again, we consider the three sequences, “city day,” “city traffic,” and “city night.” Each of the three sequences is represented by a cluster of colored dots, where each dot indicates the number of MACs used to transform a given image residual of the corresponding sequence. Spatially clustered pixels require less MACs per frame than spatially decorrelated pixels, because neighbor coefficients are updating the same coefficients at the next scale. This effect is more visible for the “city traffic” sequence with a static camera facing the street (green dots) as pixels that changed are large clusters generated by cars passing in front of the camera.

The black curve in Figure 13 illustrates the number of MACs per frame using the filter bank approach, where the changed pixels are generated in random locations, uniformly across the image. The random distribution of the pixels ensures that they are spatially decorrelated, and hence maximizes the number of MACs per frame. Thus the black curve serves as an upper bound to the number of MACs required for the frame residuals-based transform. The distance of a point to the black curve provides a measure of the randomness of the spatial distribution of the pixel changes in one frame. Points significantly below the curve are characteristic of spatial clusters of updated pixels.

By the definition of the wavelet transformation, the computation of the transformation coefficients at each scale is local and depends on the length of the high-pass and low-pass filters. At the beginning of the video sequences, background pixels are updated almost randomly, and hence the number of MACs per frame is close to the black curve. However, otherwise the trends are then different for static camera (“city traffic”) and mobile ones (“city day” and “city night”). In the “city traffic” sequence, residual images are due to spatial clusters of pixels generated by cars and pedestrians, and hence the dots corresponding to this sequence drift away from the black curve as time increases, emphasizing a lower MACs consumption per frame. In the other sequences, the background is also changing and the pixels in the image residuals appear more randomly distributed. Hence also the corresponding dots in Figure 13 are closer to the black curve. Insets in Figure 13 show samples (graylevels and residuals) of the sequences, supporting the above interpretation. We establish in Table 1 the new ratios for the three sequences comparing the event-based transform and the frame-residuals based transform. The new modified ratio is defined as:

(20) $$\begin{array}{l} R_{r}=\frac{N\left(\log_{2}^{2}\left(n\right)+3\log_{2}\left(n\right)+2\right)}{\text{total of MACs used for the residuals}}. \end{array}$$

The new ratios are closer to 1 when the transform is applied to the image residuals, as is expected. Processing only the changes between two successive frames can be seen as an extension of the event-based approach to a sequence of frames. It is interesting to observe that the “city night” sequence requires less MACs for the event-based method than for the residual-frame method, but the opposite is true for “city day.” This is an example on how the scene statistics affect the complexities of different transformation methods.

**Table 1:** Ratio of MACs used for the Haar wavelet transform between the event-based method and the filter bank method applied to images residual.

	**City traffic**	**City day**	**City night**
*R*_*r*_ (@ 1000fps)	1.10	1.45	0.89

In this work we reformulated important linear basis transformations used in signal processing into an iterative form compatible with the event-based nature of signals acquired by neuromorphic vision sensors. This event-based formulation of the basis transformations is mathematically exact, straightforward and encompasses the frame-based formulation. The main advantages of this iterative form are the signal time accuracy preservation and the minimal computation resource requirement for updating the transform when changes occur sequentially in the signal. Without need of building frames at arbitrary frequency, this event-based form allow to calculate the transformations without delay.

We have shown via natural recordings from the ATIS the performances of the “on the fly” discrete Haar wavelet transformation computation. Because the event-based signals contain low redundancy, only relevant changes in the scene are registered and processed to update the transformation output. This is beneficial for low-power real-time computation, where the computing resources can be used at maximal efficiency with respect to the desired temporal resolution.

## Author contributions

S-HI: Drafting the work or revising it critically for important intellectual content. Agreement to be accountable for all aspects of the work in ensuring that questions related to the accuracy or integrity of any part of the work are appropriately investigated and resolved. EL: Drafting the work or revising it critically for important intellectual content. RB: Final approval of the version to be published.

### Conflict of interest statement

The authors declare that the research was conducted in the absence of any commercial or financial relationships that could be construed as a potential conflict of interest.
