# Supplementary material for: Complexity Analysis of Iterative Basis Transformations Applied to Event-Based Signals
Source: Front Neurosci. 2018 Jun 12;12:373. doi: 10.3389/fnins.2018.00373 (PMC6006676; doi:10.3389/fnins.2018.00373)
Supplement: Supplementary file 1 [file Presentation_1.pdf]

Complexity analysis of iterative basis transformations applied to event-based signals

Sio-Hoi Ieng[1,*], **Eero Lehtonen**[2], **Ryad Benosman**[1]
*Correspondence:
Sio-Hoi Ieng
siohoi.ieng@gmail.com

APPENDIX

0.1 Number of non-zero elements in the Haar transform matrix

Let us consider the Haar wavelet transform matrix $H_{n\times n}$, where $n = 2^k$ for some positive integer k. Prior to normalizing its rows, the transform matrix $H_{n\times n}$ can be constructed by using the following recursive formula for $m \geq 2$:

$$H_{2\times 2} = \begin{pmatrix} 1 & 1 \\ 1 & -1 \end{pmatrix},$$
$$H_{2m\times 2m} = \begin{pmatrix} H_{m\times m} \otimes [1,1] \\ I_m \otimes [1,-1] \end{pmatrix}, \tag{S1}$$

where I_m is the $m \times m$ identity matrix, and $\otimes$ denotes the Kronecker product. We claim that each of the *columns* of $H_{2^k\times 2^k}$ contains exactly $k+1$ non-zero elements. This is proven by induction, since

1. It is true for the initial case. By construction, each of the columns of $H_{2\times 2}$ contains exactly $1 + 1 = 2$ non-zero elements,
2. Let us assume that $H_{m\times m}$ has exactly $l + 1$ non zero element per column if $m = 2^l$. By the definition of the Kronecker product and according to (S1), each of the columns of the matrix $H_{2m\times 2m}$ contains exactly one more non-zero element than $H_{m\times m}$ i.e. $l + 2$.

Hence, by induction every $H_{2^k\times 2^k}$ has exactly $k + 1$ non zero element per column. To obtain the Haar wavelet transform matrix, the rows of $H_{n\times n}$ are normalized to have the norm of 1. This does not change the number of non-zero elements.

0.2 Filter bank implementation complexity

This is the most usual implementation for the Haar transform and for discrete wavelet transforms in general, it is the most computationally efficient. The complexity is in $\mathcal{O}(n^2)$ and this can be easily seen from the bank filter structure: the signal is high-pass and low-pass filtered and then downsampled by 2. The initial dimensions of the signal are $n \times n$ and the signal resolution is divided by 2 at each downsampling i.e. at the ith downsampling, the signal dimensions are $n/2^i \times n/2^i$. The number of MACs used in the Haar wavelet transform at scale i is then equal to $2(n/2^i)^2 + 2(n/2^i)^2 = 4n^2/4^i$, where the sum terms correspond the row and the column transform. The total of MACs used for a complete transform of a $n \times n$

frame is then

$$8n^2 \sum_{i=0}^{k-1} \left(\frac{1}{4^i}\right) \underset{k \to +\infty}{\sim} \frac{16}{3} n^2, \tag{S2}$$

with $k = \log_2(n)$. The approximation in (S2) is reasonable even for small value of k (for $k = 6$, the approximation error is less than 1%).

0.3 Event-based Haar transform and the filter bank implementation

The filter bank implementation of Discret Wavelets Transforms is known for its complexity in $\mathcal{O}(n)$, for $n = 2^k$ being the length of the signal to transform in the 2D case, since we have a total of n^2 pixels, the complexity is in $\mathcal{O}(n^2)$ i.e. the complexity is linear w.r.t. the number of pixels). In the case of an event-based signal, the number of MACs required to update the transform is accounted as followed. The DWT can be decomposed into a row transform followed by a column one (or in the reverse order, this does not matter). If one single pixel changes, two new coefficients are recalculated by the low-pass and the high-pass filters at each level: this uses two MACs. The mechanism repeats for the k levels, hence a total of $2k$ MACs are necessary for the row transform and it gives $k + 1$ new coefficients. If we repeat the same reasoning for the column transform, we will account a total of $2k(k + 1)$ MACs. To summarize, one pixel change is requiring the following number of MACs for the Haar transforms implemented with the filter bank:

$$\underbrace{2k}_{\text{column transform}} + \underbrace{2k(k+1)}_{\text{row transform}} = 2k^2 + 4k \tag{S3}$$

However, this number is a upper bound. In general the number of required MACs is less because at the row transform, we are here considering that each new coefficient is consumming a pair of MACs for the calculation of new coefficients at the next scale. Actually if two adjacent new coefficients are used to recalculate new coefficient at the next scale, they are still consumming only one pair of MACs.
